# The Baby

**Published:** 1892-09

**Authors:** 


### THE BABY.

Dr. Guster contributes to a German paper the following brief but pathetic journal of a baby who, after thirteen days in this world, departed, leaving these reflections for your instructions :

First day—Wonderful, heavenly ! At last I am in this beautiful world ! Who would have thought it, that one could breathe, freely breathe, and cry out what one thinks ? I rejoice particularly in the sunlight and blue sky, in the fresh, pure air with its coolness. If I could only see and feel all this splendor !

Second day—Oh, this horrible heat ! I have been deceived. This air, this water, this light, how entirely different have I imagined it would be. But patience, all will come right by and by. The old woman who cares for me does not seem to understand me.

Fifth day—Still no solution. If it goes on this way I cannot hold out long. The whole livelong day must I lie buried in feather cushions so that I can scarcely gasp down a bit of air. Two linen and one flannel binders, a little shirt, a flannel slip, a long cushion filled with feathers, in which I am wrapped from head to foot, over this a coverlet filled with feathers, the curtains of my crib drawn to, the room darkened with double curtains, the windows closed, so must I, poor worm, lie from morning till evening. My burning skin is worse off than the hot stove near me, which can at least, as I feel, give off its heat. Oh, that I did know what I shall do. If I cry it brings the old woman with her milk, which increases my misery; my hands are cold while my brain and skin are burning, she brings a few more wraps. I turn my half closed eyes from side to side seeking help, and my tormentor says " The baby shivers," and really heats the horrible things at the stove. Will no one come to my relief ?

Tenth day—Again a fearful night ! I cry, but I am not understood. I must drink, drink, and again drink until the stomach overflows. A half hour later they give me something with a horrible taste from a teaspoon. Air, air, pure, cool air, light, water! Shall I then have no help from this world ?

Twelfth day—Yesterday there was a great council of my aunts and cousins. Each one advised a different remedy for my sickness, but all agree that its cause is a cold. Warmth was urgently recommended, and I received a new kind of infant food just discovered and some strengthening wine, which heated my brain a little more, so that I was deathly still. My body is wrapped so tightly with the roller that my stomach overflows every time a teaspoonful of any thing is given. My feet are forcibly extended and enveloped so I cannot bring them up to relieve the pain, but my feeling is gradually going. Would that all were soon over.

Thirteenth day—Farewell, thou beautiful world. Thy light and thine air have been denied me, but thither where I go there are no fetters.

